# Medical calculators derived synthetic cohorts: a novel method for generating synthetic patient data

**DOI:** 10.1038/s41598-024-61721-z

**Published:** 2024-05-20

**Authors:** Francis Jeanson, Michael E. Farkouh, Lucas C. Godoy, Sa’ar Minha, Oran Tzuman, Gil Marcus

**Affiliations:** 1 Ontario Brain Institute, Toronto, Canada; 2 Peter Munk Cardiac Centre and Heart and Stroke Richard Lewar Centre, University of Toronto, Toronto, Canada; 3 Department of Cardiology, Shamir Medical Center, Zeriffin, Israel; 4 Tel Aviv University Faculty of Medicine, Tel Aviv, Israel

**Keywords:** Interventional cardiology, Computational models, Machine learning, Predictive medicine

## Abstract

This study shows that we can use synthetic cohorts created from medical risk calculators to gain insights into how risk estimations, clinical reasoning, data-driven subgrouping, and the confidence in risk calculator scores are connected. When prediction variables aren't evenly distributed in these synthetic cohorts, they can be used to group similar cases together, revealing new insights about how cohorts behave. We also found that the confidence in predictions made by these calculators can vary depending on patient characteristics. This suggests that it might be beneficial to include a "normalized confidence" score in future versions of these calculators for healthcare professionals. We plan to explore this idea further in our upcoming research.

## Introduction

The use of synthetic patient data in medical research is rapidly gaining popularity for their ability to model large, complex multivariate datasets that replicate features of real data while maintaining patient privacy^1,2^. Several methods such as rule based methods, bayesian networks, auto-encoders, and generative adversarial networks (GANs) have gained attention for their ability to mimic real data by maintaining their statistical characteristics^3,4^. Here instead, we present an approach that unpacks the characteristics of models derived from real world patient data to not only reveal new findings from the data but also from models themselves. In particular, we apply this to medical risk calculators which are derived from real world clinical populations. Medical calculators are vital tools in clinical practice, offering a means to create measurable evidence and introduce new medical guidelines and standards. They can serve as clinical decision support systems, improving clinical efficiency and the dissemination of new medical evidence. With proper awareness and training, medical calculators can even enhance the clinical efficiency^5^. As of 2018, for instance, approximately 65% of physicians make use of the MDCalc medical calculators to inform medical decisions on a weekly basis which highlights their importance in clinical practice^6^. However, medical calculators have come under recent scrutiny as variability in their design and at-times lack of contextual insights can lead to unreliable measures^7–9^.

As part of a clinical research project focused on risks of patients that undergo a first heart attack and a recurrent one, our group developed a cohort of synthetic survivors of first heart attacks^10^. However, unlike typical synthetic data generators that focus on generating pseudo-realistic patient characteristics based on statistical assumptions and natural distributions, our clinically focused project, instead used existing medical risk calculators who themselves were based on real-world data, as the basis for generating a cohort that mapped would-be patient profiles to real-world outcomes in a near exhaustive manner. Our underlying idea is that generating synthetic patient profiles from risk calculators allows the exploration of patient cohorts in a data-driven manner without making assumptions about clinical cohort characteristics or about what data to include for model building. This is especially important considering the often limited patient characteristics that can be found from individual patient datasets at any given institution. Not only will demographics typically be unbalanced to local population features, but the medical cases observed will be influenced by other factors such as institutional specialisation, observational biases among others^11^.

Ultimately, our approach may reduce or eliminate the need for secondary analysis on clinical results or the initiation of an entirely new clinical research study. To the best of our knowledge, the creation of such a medical calculator derived synthetic cohort, and its potential and limitations for medical research, has not been previously described.

We begin by describing the methods used to create a synthetic cohort of patients having undergone an initial heart attack, population clustering identifying subgroups, and describe a novel method to complement risk scores with a confidence score of that risk we call the “normalized confidence”. We then present our results and discuss our interpretation and the opportunities we suspect this approach to leveraging risk calculators presents for future clinical research.

## Methods

### The use of medical calculators to create a synthetic cohort

The treatment given to heart attack survivors aimed at preventing future negative outcomes is called “secondary prevention” (SP)^12^. The efficacy of this SP is expected to differ between patients according to their specific health profile^13^ however there is no known way to estimate this efficacy for a given patient. The clinical study, which will be described in detail elsewhere, proposes a way to do so through the ratio between a patient’s baseline risk of undergoing a first heart attack, to their risk of undergoing a second heart attack while they are treated with SP.

Medical calculators were previously developed to estimate these two risks for a given patient, specifically the Atherosclerotic Cardiovascular Disease (ASCVD) risk calculator^14^ that provides the baseline risk for a given patient, and the Second Manifestations of Arterial Disease (SMART) calculator^15^ that provides the secondary risk. Here we use both calculators to create synthetic cohorts. It is worth mentioning that the SMART calculator is unique for predicting secondary risk, but for primary risk multiple calculators exist. The ASCVD was chosen due to its resemblance to the SMART calculator, both in input fields as well as in the type of prediction (10-year mortality risk), simplifying the calculation of the ratio between them.

### Creation of the synthetic cohort

Both ASCVD and SMART publications have references to their risk model equations and coefficients^14,15^. We began by implementing these equations in Python, and conducted validation checks to ensure their accuracy with the existing calculators. Code was also created to perform analyses, generate profiles, and store profiles in data files. We then considered each input factor in the two calculators and determined if they were categorical or numerical, we then defined bounds and intervals for numerical values. While various distributions of input factors could have been explored, we opted for a non-stochastic approach to allow for a complete exploration of the original calculators which were themselves the result of modeling population samples. This served two purposes, first this helped ensure all potential profiles are considered for clinical relevance, and second this made it explicit as to how the calculators behave on scenarios that were potentially unseen in the original patient data that were used to derive both the ASCVD and the SMART calculators. Because of the breadth of the selected uniform input space, even few variables could lead to a combinatorial explosion, making the simulation intractable practically. In our case, we found over 600 quadrillion profile combinations. If adopted, this would result in over 10 billion hours of computation on a modern processor, or over 1 million years. To achieve a tractable number of profiles without sacrificing clinical relevance, we reduced the number of factors to the core set of common risk factors between ASCVD and SMART, while also decreasing the resolution of numerical factors to clinically relevant intervals. In particular we avoided unrealistic combinations of values for these factors. We also chose to simulate only realistic profiles for patients that would have recently experienced their first heart attack.

In total, sixteen profile factors were defined based on the requirements for ASCVD and SMART. Two were used only for ASCVD, seven used only for SMART, and seven used by both. We then applied restrictions on some input factors that were dictated by the clinical scenario the clinical research question imposed (e.g., the time in years since the first heart attack variable for the SMART calculator was set to zero because the clinical research question focused on survivors of a heart attack immediately after the event). Five inputs were held at fixed value, seven were ordered variables, and the remaining were binary categorical variables. Taken together this led to a total of 26,880,000 profile combinations by applying the cartesian product on the sixteen profile factors. Table 1 shows the selected values for each factor used in the calculation of the ASCVD and SMART risk scores. Code and data are made available at the following location 10.5281/zenodo.8241872. The generation of the cohort was achieved with Python’s Itertools product combinatoric generator. All profiles were generated in approximately 1.7 h on a computer powered by an Intel Quad Core i7 with 8 GB RAM.

**Table 1:** The profile generator: the variables with used limits, intervals and rules.

Parameter	Intersected lenient with rules and mandatory choices version		
	Range of values	Number of options	Rules and implications
Age (years)	40–79, by increments of 1	40	ASCVD and SMART
Sex	Male/female	2	ASCVD and SMART
Race	African American/other (same as white)	2	ASCVD only
Systolic blood pressure (mmHg)	90–200, by increments of 10	12	ASCVD and SMART
Total cholesterol (mmol/L)	4.0–8.0, by increments of 1	5	ASCVD and SMART
HDL cholesterol (mmol/L)	0.6, 1, 1.5, 2, 2.5	5	ASCVD and SMART
History of diabetes	Yes/no	2	ASCVD and SMART
Smoker	Current/no	2	ASCVD and SMART
Coronary artery disease	Yes	1	SMART only As our cohort simulates patients after MI, with no previous history of CV disease, we chose 'yes' for history of coronary artery disease and ‘No’ for other histories
Cerebrovascular disease	No	1	
Abdominal aortic aneurism	No	1	
Peripheral artery disease	No	1	
Time since first diagnosis of CV disease (years)	0	1	SMART only As our cohort simulates patients shortly after their first MI, the answer '0' is mandatory for this parameter
Estimated GFR (mL/min)	60–120, by increments of 10	7	SMART only
High-sensitive CRP (mg/dL)	0.1, 0.2, 0.6, 1, 3, 6, 8, 11, 13, 15	10	SMART only
On hypertension treatment	Yes/no	2	ASCVD only

### Exploring the natural grouping of the cohort

As part of a separate clinically focussed research study, which seeks to adjust the risk of a cardiovascular event recurrence relative to the baseline risk of a first event, a novel measure was developed that we denote the unmet risk index (UMRI)^10^. More specifically, the UMRI is computed by dividing the secondary risk produced by the SMART score with the baseline risk produced by the ASCVD score. In this separate clinical work, we grouped patient profiles based on arbitrary thresholds determined through clinical reasoning. However, we also identified the opportunity to leverage unsupervised machine learning methods to explore these groups from the data.

Over the last two decades, computational methods to explore unsupervised methods for natural grouping of datasets via clustering have gained popularity in medical research^16–18^. Specifically, clustering methods were recently used in clinical studies of medical entities in which grouping by clinical reasoning was not feasible due to heterogeneity of the data^19,20^. We therefore wished to explore whether such natural grouping may occur in our synthetic cohort. Upfront, the unnatural uniform distribution of the variables in our cohort, due to the iterative nature of the computation of profiles (leading to 50% females, 50% diabetic, etc. See Table 2), should exclude the possibility to find natural grouping in it. However, the prediction variables of both calculators and the derived UMRI variable have a non-uniform distribution. We thus used these components of variability to explore the option of natural grouping of the cohort.

**Table 2:** Distributions of variables in the dataset.

Parameter	N (%)	Mean (SD)	Skewness	Kurtosis
Age (years)	n/a	59.5	0.0	− 1.20150
Sex—female	13,440,000 (50%)	n/a		
Race—non-Caucasian	13,440,000 (50%)	n/a		
Systolic blood pressure (mmHg)	n/a	145.0	0.0	− 1.21678
Total cholesterol (mmol/L)	n/a	6.0	0.0	− 1.3
HDL cholesterol (mmol/L)	n/a	1.52	0.08449	− 1.34334
History of diabetes—yes	13,440,000 (50%)	n/a		
Smoker—yes	13,440,000 (50%)	n/a		
Coronary artery disease—yes	13,440,000 (50%)	n/a		
Cerebrovascular disease—yes	13,440,000 (50%)	n/a		
Abdominal aortic aneurism—yes	13,440,000 (50%)	n/a		
Peripheral artery disease—yes	13,440,000 (50%)	n/a		
Estimated GFR (mL/min)	n/a	90.0	0.0	− 1.25
High-sensitive CRP (mg/dL)	n/a	5.79	0.43372	− 1.33780
On hypertension treatment—yes	13,440,000 (50%)	n/a		
ASCVD 10-year risk (%)	n/a	0.23685	1.07877	0.64918
SMART 10-year risk (%)	n/a	0.18980	1.41452	2.16970
UMRI	n/a	59.5	0.0	− 1.20150

An unsupervised clustering method was utilised to detect potential underlying groups. Because K-means clustering applies only to numerically ordered values, we adopted the K-prototypes clustering method which operates on a mix of numerical and categorical values^21^. Due to the computational load of clustering on over 26 M profiles, we applied the clustering algorithm on a uniformly random subset of 26,260 profiles. Because the optimum number of clusters is unknown, we computed the silhouette score^22^ on a series of clustering outcomes for a number of clusters between 2 to 8. Silhouette scores range between − 1 and 1, the higher scores suggest that items belong more clearly to their own group with a greater separation from other groups. Scores with values 0 or below suggest that there is very poor grouping or even overlap of vectors between groups.

### Exploring the level of confidence in risk predictions

**Figure 1:**
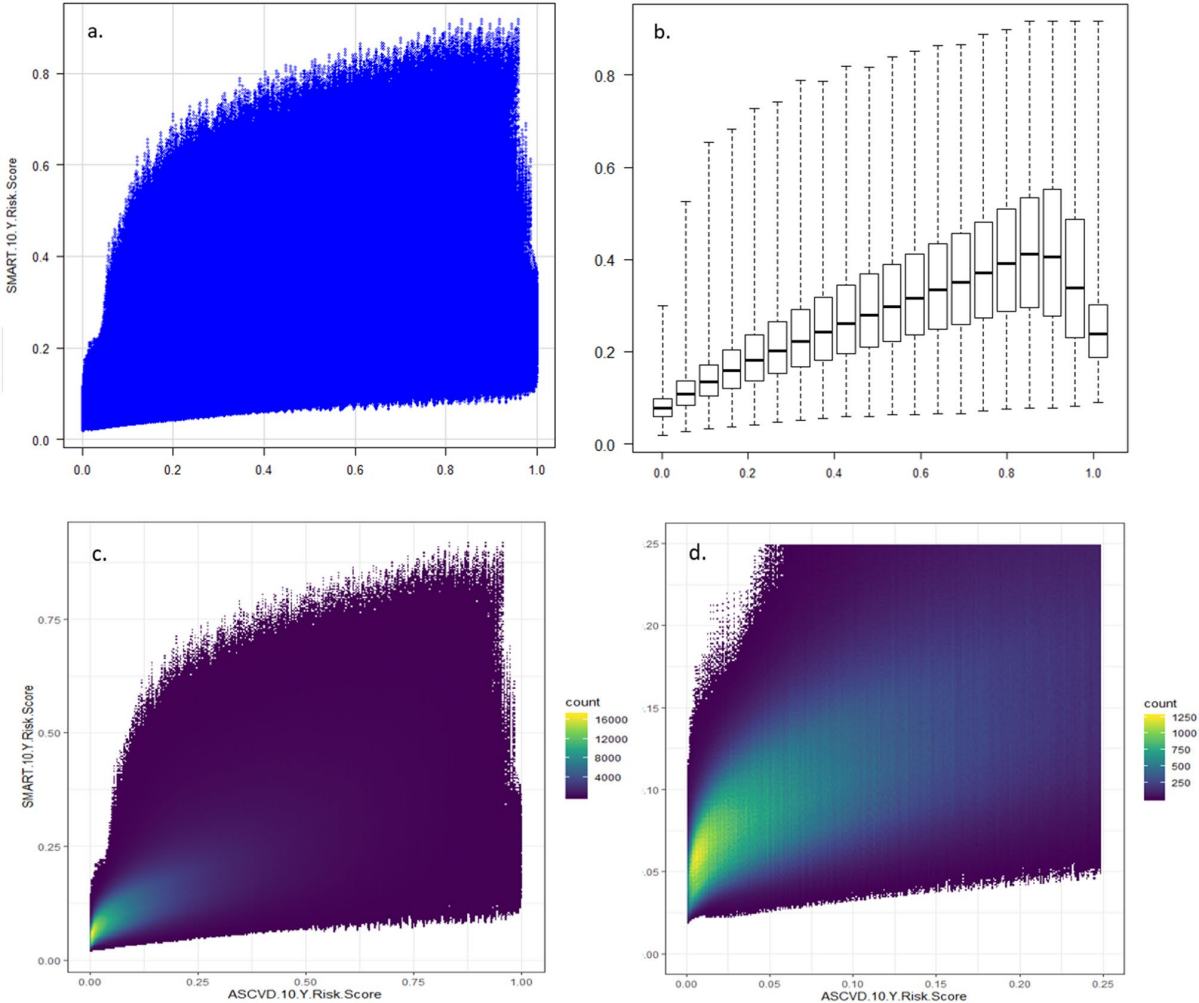
Distribution of ASCVD and SMART prediction values. (**a**) The full plot of SMART by ASCVD with all 26,880,000 profiles. Each dot represents a single synthetic patient, with its corresponding ASCVD score and SMART score. As there is overlap of dots in some areas, boxplots (**b**) and heat maps (**c**, **d**) were used to describe the distribution of the patients in the plot. (**b**) SMART by ASCVD binned into 20 equal (by width) bins. (**c**) SMART by ASCVD binned for density measurements. (**d**) Zoom-in on SMART < 25% and ASCVD < 25% from figure c.

An initial exploration of the distribution of the prediction values showed that very different profiles can lead to the same risk prediction (Fig. 1). This suggested that variability may exist in the strength of confidence of the predictions, depending on the specific profile that led to the prediction.

To explore this phenomenon, we made use of the synthetic profiles to first show the amount of variability in calculator scores. Score values for each calculator were ordered in ascending order and the standard deviation was computed on grouped scores by increments of 0.001. We then further explored variability by isolating factors and calculating the amount of variability in risk scores for each independent variable value (e.g., ‘male’ versus ‘female’ for the gender variable). By leveraging the synthetic profiles, we could compute the standard deviation of all risk scores when a single variable value is held fixed.

Using the independent standard deviation that was revealed for each risk factor, we then computed a composite measure which combines the variation found when each risk factor value is held fixed. We achieved this by dividing the standard deviation of the risk score for each isolated factor value (e.g., age = 42) by the maximum value for that factor across profiles. The product of all normalized values across factors for the risk calculator was then computed. We finally subtracted this product from 1 to obtain a score that increases as an indicator of increased confidence. This results in an overall confidence of the risk score that a calculator produces for a specific profile. We call this metric the “normalized confidence”. By normalizing variability when a single factor is held fixed and computing the inverse of the product of these variabilities, this normalized confidence score indicates highest confidence when the risk score is 1 and lowest confidence when the score is 0. The development of this score, as well as its potential application for other medical calculators to enrich clinical decision making will be presented in a separate publication.

## Results

**Figure 2:**
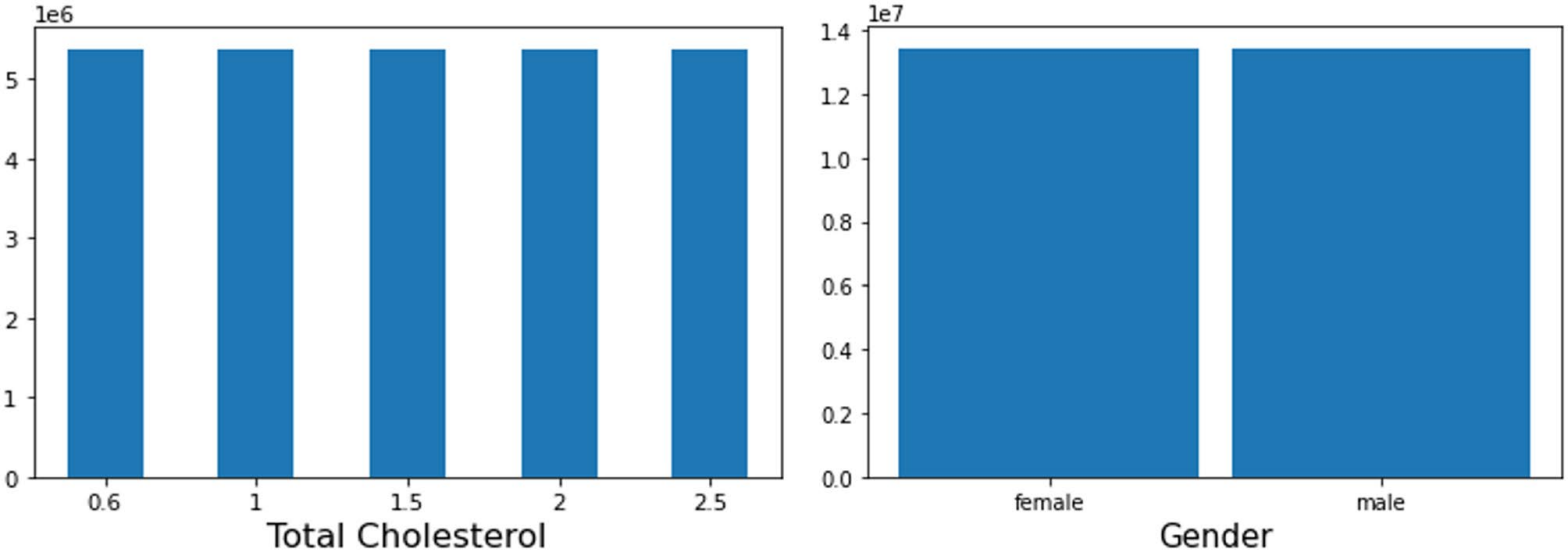
Sample diagrams to demonstrate the uniform distribution of characteristics in the computed cohort.

Basic features of our synthetic cohort are presented in Table 2 and Fig. 2. The distributions of categorical and numerical variables were uniform. In contrast, the SMART and ASCVD variables, and the resulting UMRI variable, had a non-uniform distribution.

The distribution of profiles across the ASCVD and SMART predictions is presented in Fig. 1. The highest density cluster of profiles was seen between ASCVD 0% and 2.5% risk and SMART 3% and 7% risk.

### Natural grouping of the cohort

The silhouette scores for two to eight clusters are presented in Table 3. While the overall silhouette scores remain low, the highest silhouette score was observed for two clusters, and the second highest when five clusters were defined. Clusters of size 2 will often have higher silhouette scores due to easier binary separation between groups especially if sizes become unbalanced. More interestingly, we found that while a lower silhouette score was obtained for clustering into three and four groups, a clustering into five groups led to a slightly higher silhouette (0.2957) only to drop again at higher cluster numbers. This might suggest that five prototypical centroids may best describe the natural groups in the synthetics profiles when risk scores are included. While the contribution of scores may be the largest factor for item cluster assignment, clustering enables us to identify other common properties that associate with each grouping.

**Table 3:** Silhouette scores for a random subsample of size 26,260 for a number of clusters C between 2 and 8 on numerical values only.

**Silhouette score with C = 2**	**0.41810**
Silhouette score with C = 3	0.28986
Silhouette score with C = 4	0.28873
**Silhouette score with C = 5**	**0.29571**
Silhouette score with C = 6	0.28879
Silhouette score with C = 7	0.27571
Silhouette score with C = 8	0.25487

Significant values are in bold.

Some grouping was found by the K-prototypes clustering algorithm. The low silhouette scores below 0.5 suggest that some separation, with limited distinction, was found between the features of the profiles which included the UMRI score. A high score for C = 2 is typically found when low dimensionality suggests easier separation between groups. However, a noticeable second-peak silhouette score was found when 5 clusters are defined, whereas lower values are found for C = 3 and C = 4 as well as for C = 6 and above. This suggests that higher dimensional and more balanced grouping was found for clusters of size 5.

**Figure 3:**
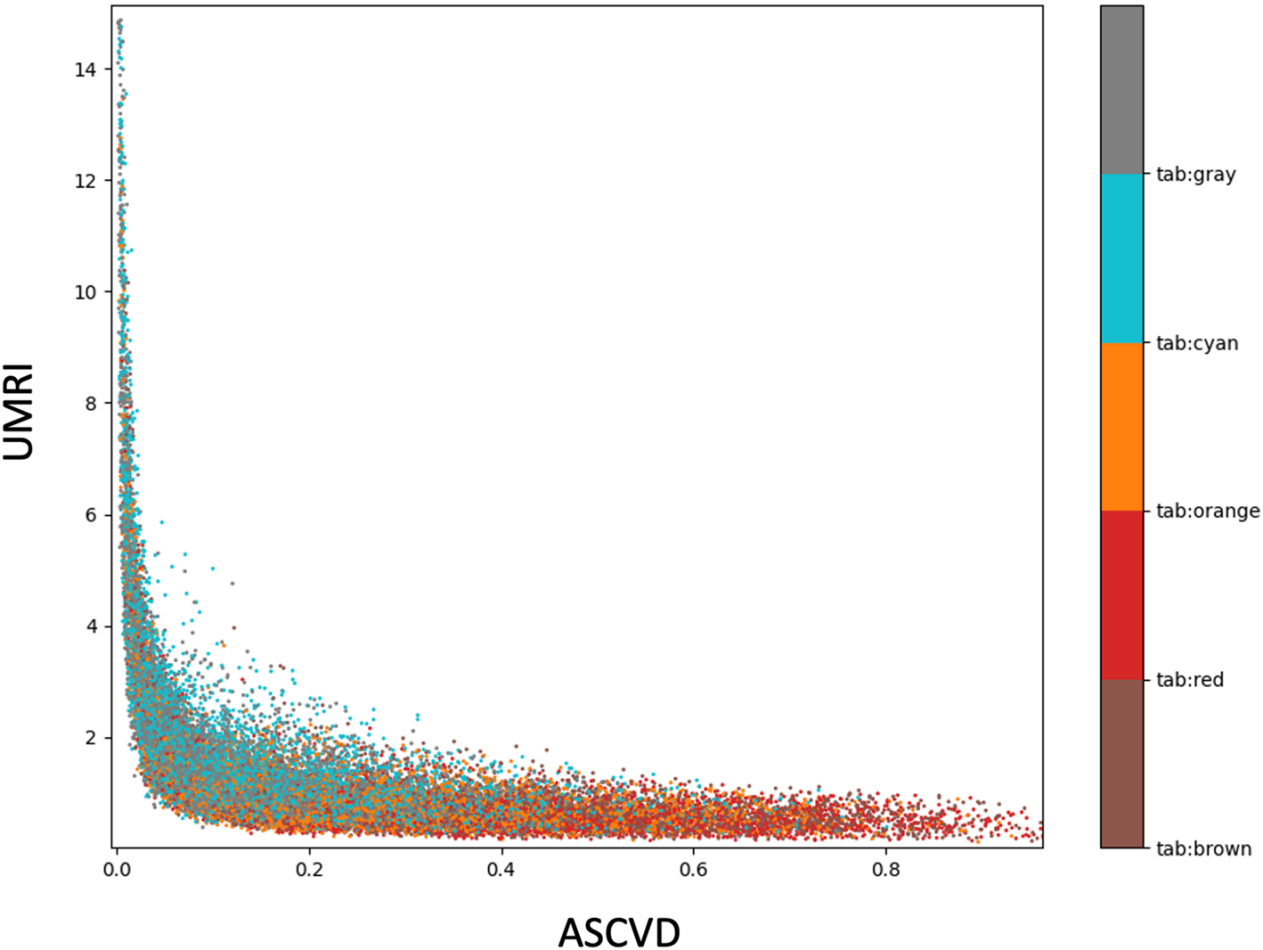
K-prototype clustering for a target of 5 clusters. K-prototype clustering for a target of 5 clusters on all profile generating input categorical and numeric variables, as well as the post-hoc UMRI score. UMRI was cropped at 15 to illustrate details of the membership categories represented by either of the five colors.

Figure 3 shows the cluster membership among the five clusters, across the ratio between the calculators. In general, the groups clustered along the transition from high UMRI to low UMRI (the y-axis), as well as from low ASCVD to high ASCVD (the x-axis), in a similar way to the grouping done by the clinical arbitrary cut-offs between groups. However, in contrast with the clinical grouping and despite this general similarity, some outliers can still be identified which reach the tail-end of the cluster when viewed as a relationship between ASCVD and UMRI (i.e., red-dotted profiles that are more densely found on the right-side of the graph can nevertheless be found in lower numbers on the left-sided side). This indicates that very similar profiles (with the same colour) may have very different baseline and/or secondary risks.

A secondary study is planned to understand if there is a clinical meaning for these groupings, as well as a specific search for the differentiating factors with two similar profiles with unsimilar risk predictions.

### Level of confidence in output variables

**Figure 4:**
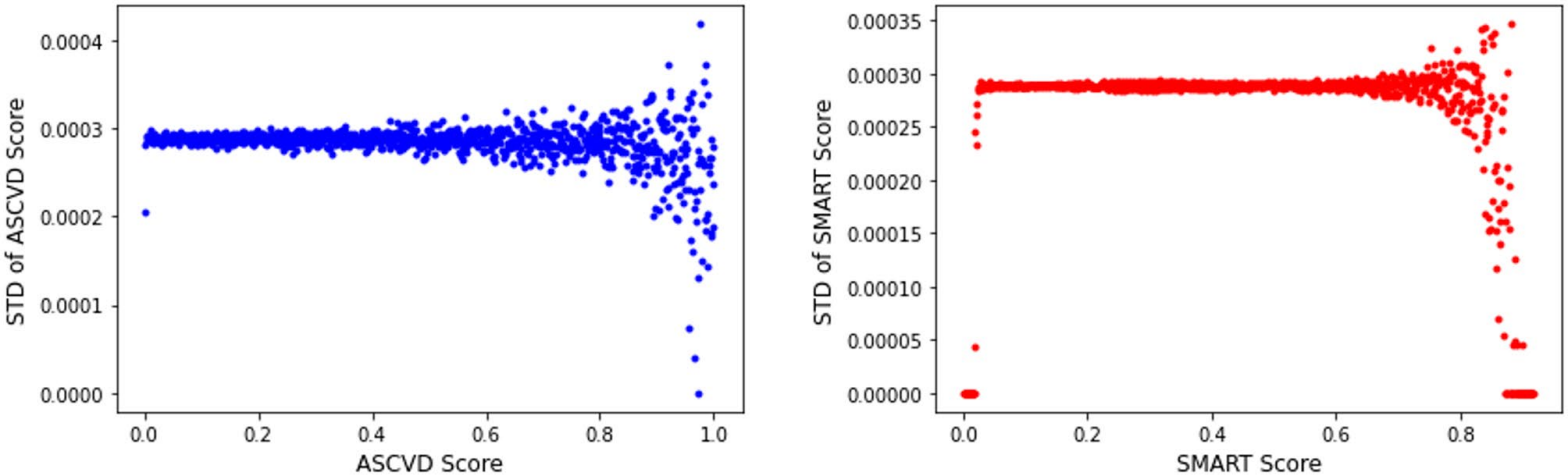
Standard deviations of ASCVD and SMART as the risk scores increase. Standard deviations of grouped risk scores for ASCVD and SMART as risk scores increase by increments of 0.001.

**Figure 5:**
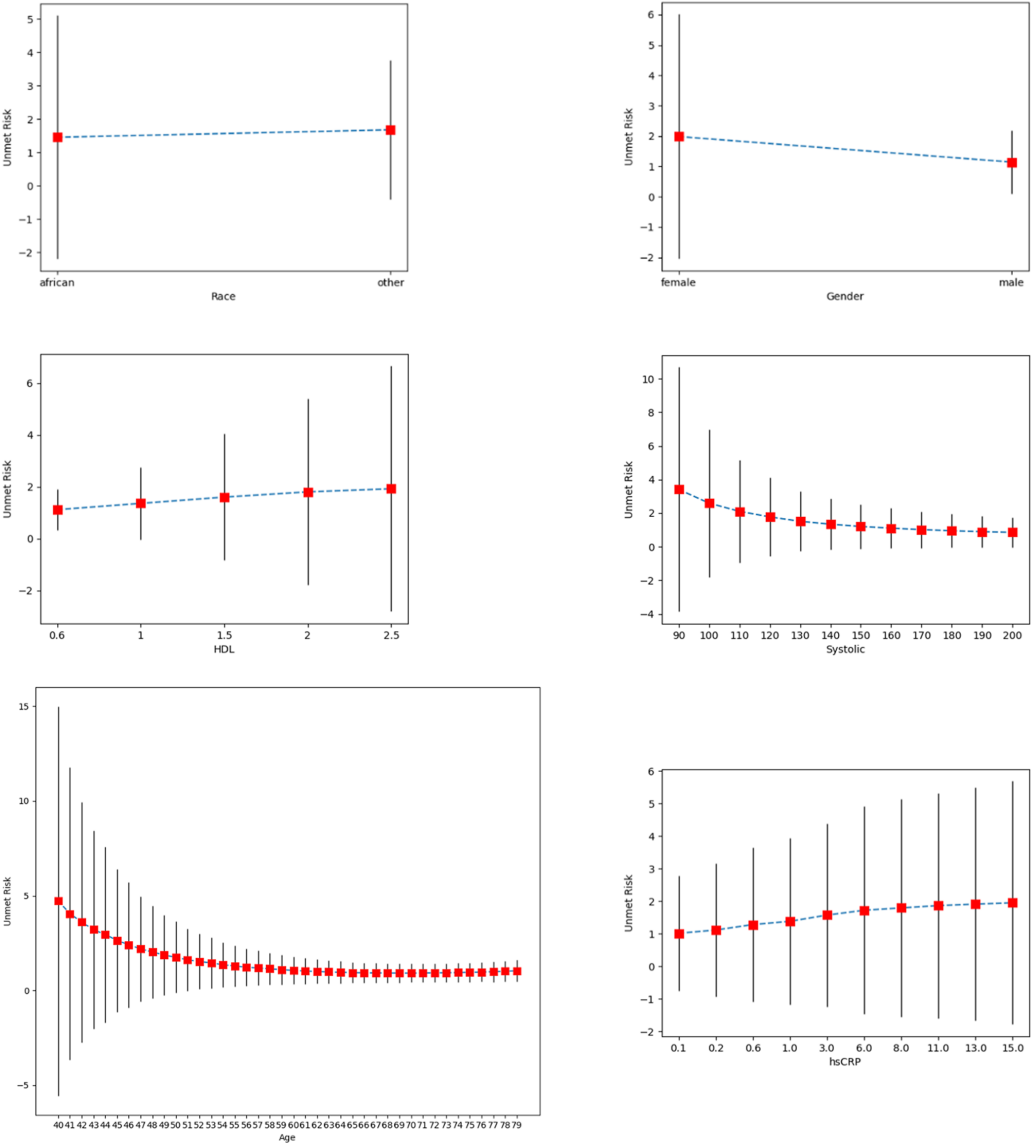
Mean Unmet Risk Index with standard deviations for select variables. Mean Unmet risk index (UMRI) for all “African” and “Other” race profiles; “Female” and “Male” gender profiles; for age profiles; for systolic blood pressures; for high-density lipoprotein (HDL), and for high-sensitivity C-reactive protein (hsCRP). Red square show means, vertical line show ± 1 standard deviations.

Figure 4 shows that as the ASCVD and SMART scores increase, the standard deviations of each of these scores computed tend to increase in variability in a geometric way as risk scores increase. The variability of key categorical and numeric variables is presented in Fig. 5. Significant differences in variance were observed for race (SD 3.65 for ‘African’ vs. SD 2.1 for ‘other’), and sex (SD 4.03 for ‘female’ vs. SD 1.04 for ‘male’). A log-like decrease was observed in variability of the risk calculators as ages increased from 40 years old (SD 10.27143) to 79 years old (SD 0.57655). Similarly, large changes in UMRI scores were found for decreased systolic blood pressures, increase in HDL levels, as well as increase in hsCRP levels.

We found that diverse profiles can lead to similar risk scores. Table 4 presents three examples of pairs of profiles with similar ASCVD scores rounded at the tenth of a percentile, with their corresponding normalized confidence. As seen with these examples, profiles with 7 or more differences with respect to risk factors, with less than 0.5% differences in ASCVD scores, can still exhibit a normalized confidence with a differential above 40%.

**Table 4:** Example profile pairs with 7 or more differences in risk factors, with ASCVD difference below 0.5% and normalized confidence differences greater than 40%.

Race	Gender	Age	Systolic	Cholesterol	HDL	Diabetes	Smoker	On hypertension treatment	ASCVD risk score	Normalized confidence
Other	Male	54	100	8	0.6	No	No	No	0.11176	0.88088
Other	Female	42	190	8	2.5	Yes	Current	Yes	0.11443	0.44133
Other	Female	40	200	8	2.5	Yes	Current	Yes	0.11859	0.41265
African	Male	60	140	5	0.6	No	No	No	0.1186	0.81831
Other	Male	76	110	5	2.5	No	No	Yes	0.19499	0.82875
African	Female	40	190	7	2.5	Yes	Current	Yes	0.19523	0.41984

## Discussion

We described the creation of a synthetic cohort from an iteration of clinical calculators—a cohort that was subsequently used in a clinical study that will be presented elsewhere. We also showed that such a synthetic cohort, despite its simulated nature, can be used to generate new medical insights. We demonstrated this in several different ways.

First, we showed that when the correlation between two risk estimations, i.e., the outputs of two medical calculators, is the focus of interest of a clinical study (as was the case in our clinical study)—the iteration of the two calculators is useful to reveal the full picture of the correlation, that by itself led to further insight by clinical reasoning, grouping, and analysis of the dataset.

Second, we showed that despite the expected unnatural uniform distribution of variables in such an iteration-derived synthetic cohort, the existence of the non-uniformly distributed prediction variables added a degree of variability that was sufficient to allow for unsupervised clustering to find natural groupings. While no strong separation was found between cluster sizes, the presence of separation elicits further investigation. Exploring cohorts according to their naturally occurring grouping may lead to a different understanding of their behaviour and thus to a different clinical insight than those found in clinical studies. Indeed, computing challenges still remain with cluster analysis on very large cohorts.

Third, by creating an extensive list of patient profiles that span into the tail-ends of the natural distribution used when the risk calculators were first created, we tested the limits of their accuracy. By exploring the effect of individual parameters on the risk we demonstrated that variability exists in the degree of confidence the calculators have in their prediction. We further showed that while this confidence variability generally increases with the overall risk, it is the specific combination of patient attributes that determine the confidence in the risk—which we can compute with this method. This information may prove valuable to clinicians who use and trust calculators for decision making. In particular, we believe that versions of calculators that will include the “normalized confidence” score could potentially benefit clinical users by providing not only a calculated risk but also a level of confidence of this calculated risk. We plan to explore this concept and its implications in subsequent work.

Conducting clinical studies is often cumbersome and costly on human and economic resources^23^. With increased computing capabilities and our ability to process large datasets, there is much interest in the option of generating new clinical insight from data that were previously collected. Usually, this is done by using different tools to explore real data that were collected in previous studies. Here instead we propose a different model. Although calculators are generated from real data collected by the recruitment of a large number of participants, they can make predictions on a much broader population of profiles than they were originally created from. In effect, generalizations are routinely made in the clinic with risk calculators in a trustworthy manner. On one hand, trusting insights generated from a synthetic cohort mapped to risk calculator results is thus justifiable as a result. On the other hand, further exploration of the performance of the calculator on previously unseen profiles in the original study is justified as well.

It is important to emphasize that the cohort created here contains an unnatural distribution, in which each profile appears exactly once, and that rare (in real-life) profiles are as frequent as common (in real-life) profiles. This precludes any attempt to draw conclusions about the prevalence of profiles in the population or the effect of naturally distributed cohorts which other models may seek to achieve. Instead, our approach helped illustrate that many possible profiles lead to similar predictions in ways that natural stochastic distributions might not. In real life these many profiles may well be very rare. Others may choose to apply the methods we showed to create a cohort from one or a combination of more than one calculator using more naturalistic stochastic distributions to achieve different research objectives. One might add the skewed distribution of the predictions of the calculators, or their product, to the set of characteristics to achieve this. In addition, profiles that have a statistically very low probability of existence could be removed. In cases where a more normally distributed and complete set of profiles are created, as would be the case in a clinical study, the correlation between the predictions of the calculators for a single profile may be the focus of interest, regardless of this profile’s prevalence in real life. That said, after demonstrating the UMRI concept with the synthetic cohort, we plan to conduct a clinical study that explores its utility with a real cohort of heart attack survivors.

In conclusion, health risk calculator derived synthetic cohorts are a potential substrate for research, when the inquiry in question is suitable to its attributes and limitations. We believe the methods presented here may be of use to others to conduct such studies in other fields. Future projects will clarify the potential of these methods.

## Data Availability

The 26,880,000 synthetic profile combinations containing computed ASCVD, SMART, and UMRI scores along with their required input values were compiled into a comma separated values (CSV) file named “umri_profiles_full.csv”. The zipped version of this file is made available under CC 4.0 attribution at 10.5281/zenodo.8241872. This data file was used to compute all statistics, clustering, and normalized confidence calculations in the analyses presented herein.

## Code availability

The code used to generate profiles as well as to compute statistics, plots, clustering, and normalized confidence scores is also available on GitHub by a link made available on Zenodo at 10.5281/zenodo.8241872. ASCVD model coefficients are available in the following manuscript: 10.1161/01.cir.0000437741.48606.98. SMART model coefficients are available in the following manuscript: 10.1136/heartjnl-2013-303640.
